# The Current Concept of T _H_ 17 Cells and Their Expanding Role in Systemic Lupus Erythematosus

**DOI:** 10.1155/2011/810649

**Published:** 2011-03-22

**Authors:** Daniel Perry, Ammon B. Peck, Wendy C. Carcamo, Laurence Morel, Cuong Q. Nguyen

**Affiliations:** ^1^Department of Pathology, Immunology, and Laboratory Medicine, University of Florida, Gainesville, FL 32610, USA; ^2^Department of Oral Biology, College of Dentistry, University of Florida, 1600 SW Archer Road, P.O. Box 100424 Gainesville, FL 32610, USA; ^3^Center for Orphan Autoimmune Disorders, College of Dentistry, University of Florida, 1600 SW Archer Roadd, Gainesville, FL 32610, USA; ^4^Eli and Edythe L. Broad Institute, 7 Cambridge Center, Cambridge, MA 02142, USA; ^5^Department of Chemical Engineering, Massachusetts Institute of Technology, 77 Massachusetts Avenue, E25-545, Cambridge, MA 02139, USA

## Abstract

Systemic lupus erythematosus (SLE) is a chronic autoimmune disease with a multifaceted range of symptoms affecting almost every organ system. The prototypical pathology of SLE involves the production of antinuclear antibodies and the deposition of immune complexes in basement membranes throughout the body where they induce inflammatory responses. The genetic and environmental etiologies of this process are being intensively sought, and recently, T_ H _17 cells have been implicated in the pathogenesis of SLE. T_ H _17 cells are CD4+ memory T cells that behave as both helper and effector cell populations functioning through their signature IL-17 cytokines. Their differentiation is distinct to either the T_ H _1 or T_ H _2 cell lineage, but strongly influences development of adaptive responses, including autoimmunity. This paper details the biological functions and regulation of T_ H _17 cells, followed by an update of their expanding role in SLE.

## 1. Introduction

The vertebrate immune system has evolved to protect its host against invading pathogens and other environmental antigens. It is strategically organized to optimally guard against foreign or “nonself” antigens through intricate interactions between innate and adaptive immunity, allowing for the survival of the host. The adaptability and resiliency of the immune system rely on complex physiological and immunological mechanisms, many of which remain to be unraveled. Since the initial classification of T_H_1 and T_H_2 cells by Coffman, Mosmann, and colleagues in 1986, much focus has attempted to elucidate the role of helper T cell populations. These efforts have led to the identification of a distinct T helper population, called T_H_17 cells [1–3], which challenges the long-standing T_H_1/ T_H_2 paradigm and has advanced our overall understanding of T helper cells in health and disease.

Paradoxically, the same mechanisms that prevent disease quite commonly induce hypersensitivity and autoimmunity. In fact, it was in the study autoimmunity in which the key observations that led to the discovery of T_H_17 cells were made. These studies found that T_H_1 cells were not required for induction of experimental autoimmune encephalomyelitis (EAE) in mice, as had been thought [4, 5]. EAE induction instead required an IL-23-dependent set of T cells that were later identified as the unique T_H_17 cell subset. Since then, numerous reports have shown T_H_17 cells to be relevant, and sometimes central, to autoimmune pathogenesis, highlighting them as therapeutic targets. Recently, T_H_17 cells have been implicated in SLE pathogenesis. SLE is a chronic inflammatory disease characterized by autoantibodies to nuclear antigens. It can be difficult to diagnose and to treat due its multifaceted nature, and death usually occurs due to renal involvement. In this paper, we discuss the biological function and regulation of IL-17 and T_H_17 cells. We will then focus on our current understanding of the role of T_H_17 cells in murine and human SLE.

## 2. IL-17 and T_H_17 Cells

This subset of CD4+ memory effector T cells is functionally distinct from either the T_H_1 or T_H_2 cell lineage [6–10]. T_H_1 cells release mainly IFN-*γ* and TNF-*α* that regulate cell-mediated immunity through activation of macrophages, NK cells, and CD8^+^ T cells. This process is driven by IL-12 through signal transducer and activator of transcription 4 (STAT4) activation and results in the expression of the transcription factor T-bet. T_H_2 cells predominantly produce IL-4, IL-5, and IL-13. IL-4 regulates the humoral immunity through the activation of B lymphocytes. The process is driven primarily by the phosphorylation of STAT6 resulting in the activation of transcription factor GATA binding protein 3 (GATA-3). Unlike T_H_1 and T_H_2 cells, differentiation of T_H_17 cells *in vitro* is mediated by TCR signaling in the presence of TGF-*β* and IL-6 or IL-21 stimulation [8]. Although IL-23 is not required for differentiation of T_H_17 cells, it is necessary for their survival and maintenance [11]. Temporal expression analysis of IL-23R indicated that it is only expressed after activation of naïve T cells with TGF-*β* and IL-6. Therefore, its expression allows for the continuous stimulation of the differentiated cells. T_H_17 effector cells are characterized by the unique ability to secrete IL-17A and IL-17F in response to stimulation by TGF-*β* and IL-6. 

At present, there are multiple factors that are known to contribute to the development of T_H_17 cells. The main regulator of T_H_17 differentiation is the T-cell-specific *γ* (ROR*γ*t) transcription factor induced by IL-6 and TGF-*β* [12, 13]. In addition to ROR*γ*t, other transcription factors also play critical role in T_H_17 cells-specific lineage development. A recent study has indicated that I*κ*Bz works in conjunction with ROR*α* and ROR*γ* in the absence of IL-6 and TGF-*β* could optimally induce T_H_17 cell development. Elimination of the transcriptional activation domain as well as the ankyrin repeat domain in I*κ*Bz would abolish its function in inducing T_H_17 cell formation through the downregulation of the NF-*κ*B pathway. I*κ*Bz physically interacts with the noncoding sequences 2 (NCS 2) regulatory element in the *Il17a *promoter region to enhance *IL17a* gene expression [14]. Although no specific mechanism was proposed, a study by Schraml et al. suggested that the activator protein (AP)-1 protein B-cell-activating transcription factor (BATF) regulates the development of T_H_17 cells by interacting with target genes downstream of IL-6 and TGF-*β* signaling. These downstream genes include the conserved intergenic elements in the *Il17a-Il17f* locus and to the *Il17, Il21*, and *Il22* promoters regions [15]. In addition, another transcription factor such as IRF4 is also involved in T_H_17 cell development. IRF4 is mediated by IL-21 to physically bind with the *Il17* promoter and act in conjunction with ROR*γ*t for optimal IL-17 transcription. IRF4 is also involved in the balance of Foxp3, ROR*α*, and ROR*γ*t during T_H_17 cells differentiation [16]. Furthermore, otherfactors which belong to the Runx transcriptional factor family member could regulate the generation of T_H_17 cells. The family includes Runx 1, Runx 2, and Runx 3; however, only Runx 1 appears to play a more specific role in promoting T_H_17 cells. An *in vitro* study showed that overexpression of CD4+ T cells with Runx 1 resulted in higher IL-17 production in the presence of TGF-*β* alone and more enhanced in IL-17 level when stimulated with both IL-6 and TGF-*β*. Therefore, the activation of ROR*γ*t by TGF-*β* alone or combination of IL-6 and TGF-*β* along with the overexpression of Runx 1 allowed for optimal T_H_17 cells formation. Using chromatin immunoprecipitation or ChIP assay, the authors demonstrated that the enhanced level of IL-17 was to due to the recruitment and synergistic binding of ROR*γ*t and Runx 1 to the *Il17* promoter and the CNS-5 enhancer region [17]. Interestingly, Foxp3 can inhibit Runx 1 and ROR*γ*t to promote regulatory T cells (Treg). Therefore, the transcriptional regulation and the dynamic interaction of these factors provide more complexities in understanding the development of T_H_17 cells. These interactive factors need to be considered when attempting to categorize different T cell populations. 

The IL-17 family of cytokines consists of six members: Il-17A (referred to as IL-17), IL-17B, IL-17C, IL-17D, IL-17E (IL-25), and IL-17F. Detailed descriptions of each cytokine, in addition to IL-21 and IL-22 which are also produced by T_H_17 cells, will be discussed below. 

### 2.1. IL-17A and IL17-F

Currently, IL-17A and IL-17F are the best characterized cytokines within the IL-17 family. IL-17A and IL-17F exist either as homodimers or as IL-17A/IL-17F heterodimers [18]. Receptors for IL-17A and IL-17F include IL-17RA and IL-17RC [19–21]. Activation of IL-17A and IL-17F initiates powerful inflammatory responses and further induces production of potent proinflammatory cytokines. Both IL-17A and IL-17F can mediate the production of IL-6, CCL3, and G-CSF in macrophages, but only IL-17A can activate CCL2, IL-1*β*, IL-12p70, and IL-9. IL-17A is also solely responsible for the activation of CCL2, CCL3, GM-CSF, IL-1*β*, and IL-9 in CD4+ T cells [22]. As part of the local inflammatory response, both cytokines are responsible for the proliferation, maturation, and recruitment of neutrophils [1]. They provide immediate immunological protection by producing antimicrobial and acute phase response proteins against a variety of pathogens, specifically *Propionibacterium acnes*, *Citrobacter rodentium*, *Klebsiella pneumoniae*, *Bacteroides spp*., *Staphylococcus aureus *[23], acid-fast *Mycobacterium tuberculosis*, and fungi infection such as *Candida albicans *[18, 24]. 

Most importantly, having the potential to upregulate the expression of specifics matrix metalloproteinases (MMPs) such as MMP-1, MMP-3, MMP-9, MMP-13, IL-17A, and IL-17F have been shown to be tissue-damaging cytokines and are intimately involved in autoimmune diseases, for example, Crohn's disease [25, 26], EAE [4], collagen-induced arthritis (CIA) [5], Sjögren's syndrome (SjS) [27, 28] and SLE which will be later discussed [29–34]. However, a recent study by Ishigame et al. [23] suggested that there are differential roles for IL-17A and IL-17F in autoimmune responses, in which IL-17F played a minimal role in the pathogenesis of delayed-type and contact hypersensitivities, EAE, CIA, and arthritis in animal models. In contrast, IL-17A appeared to produce more potent pathogenic cytokines in macrophages, whereby genetic knockout of *il-17a *rendered the mice with reduced disease phenotypes. The differential role of IL-17A and IL-17F raises interesting questions in deciphering mucosal immunity and autoimmunity. Both cytokines elicit their responses via similar receptor complexes; however, it is intriguing that they provide different autoimmune responses in terms of pathogenicity and protection. The contrasting biological functions could be due to an approximate 10-fold more potent induction of cytokines by IL-17A as compared to IL-17F [35]. In support of this concept, a recent review by Dubin and Kolls [36] has suggested a model which emphasizes the bioactivities of these cytokines on myeloid versus nonmyeloid cells or macrophages versus CD4+ T cells discussed priorly. Therefore, it is the ability of IL-17A to induce stronger responses and affect a wider range of cellular targets, making it a more pathogenic cytokine. It will be of interest if such a dichotomy is seen in SLE.

### 2.2. IL-17B, IL-17C, and IL-17D

IL-17B, IL-17C, and IL-17D are the least studied members of the IL-17 cytokine family. It remains speculative whether they are capable of eliciting any proinflammatory or protective responses like IL-17A and IL17F. A study using the CIA mouse model has shown that adoptive transfer of IL-17B+ or IL-17C+ CD4+ T cells was able to recapitulate a CIA phenotype and that blockade of IL-17B prevented disease exacerbation. The authors suggested that the inflammation induced by IL-17B/IL-17C is mediated by the production of TNF-*α* [37]. However, genetic association study in coeliac disease (CD) using large sample of patients and controls (409 CD, 355 controls) provided no conclusive evidence in the association of the genetic variation of a number of cytokines including IL-17B and the development of the disease [38]. Interestingly, our recent microarray data from the C57BL/6.NOD-*Aec1Aec2* mouse model of primary-SS revealed a strong upregulated expression of *Il17b,* thus, this cytokine may play an important but unidentified role in the rheumatic diseases (unpublished data).

### 2.3. IL-17E (IL-25)

Designated as IL-25, IL-17E has been shown to induce T_H_2-like responses with the upregulation of IL-4, IL-5, and IL-13 gene expression [39]. Furthermore, the ability of IL-17E to promote the expression of adhesion molecules, specifically ICAM-1, ICAM-3, and L-selectin, allows for eosinophilic infiltration and structural changes of epithelial cells, making it a vital cytokine for allergic inflammatory response and/or asthma-related attacks [40]. Activation of T_H_2-related cytokines also resulted in significant elevation of IgE, IgG_1_, and IgA levels in which IgE can induce the release of prostaglandin D_2_ (PGD_2_) on mast cells that directly mediates the vasodilatation, mucus production, and broncho-constriction. While IL-17E is normally negatively regulated by a Socs3-dependent pathway [41], numerous approaches have attempted to inhibit the biological function of IL-17E. Administration of monoclonal antibody that blocked the function of IL-17E dramatically reduced the production of IL-5/IL-13, infiltration of eosinophils, and IgE secretion, thereby preventing the antigen-driven airway inflammation and airway hyperresponsiveness (AHR) [42]. Taking advantage of the ability of IL-17E to bind to IL-17RA and IL-17RB receptors, antagonist monoclonal antibodies against either IL-17RB or IL-17RA receptor resulted in complete abolishment of IL-17E-induced AHR in naïve BALB/C mice [43]. A better understanding of IL-17E has shed additional information on the immunological activities of IL-17E cytokine and its participation in allergic inflammation, thereby providing potential therapeutic targets. Interestingly, a recent study by Kleinschek et al. [44] has indicated that IL-17E might play an opposing role to IL-17A. Knocking out IL-25 rendered the animals highly susceptible to the development of EAE characterized by the increase of IL-23 level and infiltration of IL-17 and IFN-*γ* producing T cells in the central nervous system. Furthermore, neutralization of IL-17A in the knockout mice prevented EAE [44]. This data clearly suggests the inhibitory role of IL-17E in EAE. However, genetic association study in patients with Crohn's disease or ulcerative colitis concluded that IL-17E has little association in the disease development [45]. Consequently, extensive studies are needed to fully elucidate the role of IL-17E in human disease.

### 2.4. IL-21 and IL-22

In addition to their signature cytokines, T_H_17 cells also produce IL-21 and IL-22. IL-21 functions as an autocrine cytokine which allows for an alternative differentiation pathway for T_H_17 cells when IL-6 is absent [46]. Furthermore, IL-21 is involved in the amplification of T_H_17 cell-specific lineage transcription factors allowing for the maintenance and stabilization of this cell population [16, 47]. IL-21 is also known to assist the activation and differentiation of naïve B cells to plasma cells by upregulation of Blimp-1 [48]. In addition, it induces the expression of the *γ*1 and *γ*3 germline transcripts for the isotypic switching to IgG1 and IgG3 from IgMin human B cells [49]. These features, thereby, establish IL-17-producing cells as helper T cells. The isotypic switching potential of IL-21 is critical in modulating the disease development of isotypic-dependent autoimmune diseases such as SLE [50] and SjS [28, 51–54]. In the nonobese diabetic (NOD) animal model for SjS, isotypic switching to an IgG1 antibody against the acetylcholine receptors (AchRs), specifically the muscarinic receptor type 3 (M3R), is required for the development of SjS. Perhaps, the most critical is its involvement in the formation of germinal centers by controlling the expression of Bcl-6, which regulates the survival and activation of B cells. Furthermore, IL-21 is necessary for the expansion of T_H_17 cells and follicular T helper cells through the costimulatory ICOS and c-Maf pathway [55]. Therefore, it is a critical cytokine in modulating not only the T cell biology, but also the B cell response. 

IL-22 is a cytokine that is produced by subsets of T_H_17 cells as well as a multitude of other cell types, including natural killer cells-22 (NK-22), lymphoid tissue inducer (LTi) cells, and epithelial cells. Mucosal microflora can promote the secretion of IL-22 from epithelia and the differentiation of IL-22-producing cell populations, in particular cell populations expressing NKp46, for example, the ROR*γ*t+CD3−NKp46+ NK cell, the ROR*γ*t+CD3−NKp46− LTi cell, and an uncharacterized ROR*γ*t+CD3+NKp46+ cell population. The IL-22 receptor complex is a heterodimeric molecule composed of IL-22RA1 and IL-10R2 [56]. On interacting with its heterodimeric receptor (IL-10R2/IL-22R), IL-22 can transduce a signal through phosphorylation of tyrosine kinases Jak1 and Tyk2, followed by the activation of STAT3, and to a lesser degree a heterodimeric STAT1/STAT3 during signaling cascade [57]. IL-22 has also been reported to activate several signaling pathways, including the MAPK pathway via ERK1/2, JNK, and p38 for induction of IL-22-related genes [58]. Since epithelial cells express high levels of IL-10R2 and IL-22R, IL-22 can initiate a strong response from epithelial cells which includes production of cytokines, chemokines, acute phase proteins, and a number of antimicrobial molecules such as *β*-defensin, lipocalins, and calcium binding S100 proteins [59]. It is also involved in tissue repair following exacerbated immune responses and epithelial-barrier functions against bacterial infections [60]. Paradoxically, IL-22 has been shown to be pathogenically associated with several autoimmune diseases including rheumatoid arthritis [61] and Crohn's disease [62] as well as non-autoimmune diseases such as respiratory-distress syndrome [63] and cystic fibrosis [64]. Whether IL-22 is an important player in development and/or onset of rheumatic disease, like SLE, will be an interesting area of future studies.

## 3. Negative Regulation of T_H_17 Cells

It remains controversial whether T_H_17 cells are protective or pathogenic. Its mode of response is substantially dependent on the eliciting antigenic entities. In certain cases of fungal and bacterial infections, IL-17 can be protective by recruiting neutrophils to the site of injury; however, IL-17 activation can also lead to rampant and impetuous immune response resulting in exacerbated clinical pathology and autoimmunity. Therefore, regulatory elements of the IL-17/T_H_17 system are required to maintain congruency and homeostasis between the protective and pathogenic consequences. Although the research area is still in its infancy, as of present, there are clearly multiple systems that have the capability to regulate the development and differentiation of T_H_17 cells. One of the most critical regulatory factors is the IL-27 cytokine, which is secreted by activated macrophages and dendritic cells [65]. IL-27 is a member of the IL-12 family of cytokines and is comprised of a heterodimer between IL-27*α* (IL-27 p28) and IL-27*β* (IL-27 Ebi3) [66]. IL-27 (or the IL-27 p28 subunit *per se*) exerts the IL-27-associated biological effects by activating its heterodimeric IL-27R including WSX-1 and gp130. Signal transduction involves phosphorylation of JAK1, JAK2, STAT1, STAT3, STAT4, and STAT5 in T cells, NK cells, and monocytes, but only STAT3 in mast cells [67]. However, only STAT1 or STAT3 activation is critical for the resulting bioactivity of IL-27 on naïve T cells that express IL-27R [68]. Activation of STAT1 by JAK1 or JAK2 promotes T_H_1 differentiation via the upregulation of T-bet resulting in the production of IFN-*γ*. At the same time, IL-27 inhibits the production of IL-2 and IL-6, thus downregulating the IL-6-dependent STAT3 activation of ROR*γ*t expression and subsequent development of T_H_17 cells. Recent studies have suggested that IL-27 is pleiotropic, regulating hematopoietic stem cell differentiation, eliciting antitumor activities, as well as promoting both pro- and anti-inflammatory activities [69–72]. Due to its potent suppressive ability, IL-27 functions to inhibit the differentiation of T_H_17 cells in both *in vitro* and *in vivo* studies. In several animal models of autoimmune diseases, a deficiency in either IL-27 or IL-27R results in exacerbated pathology and clinical signs mainly due to the dysregulation and increase in numbers of IL-17 producing T cells [11]. Additionally, systemic injection of rIL-27 cytokine into autoimmune animal models of EAE, scleritis, or uveitis ameliorates many clinical symptoms [73]. Thus, the T_H_17/IL-23/IL-27 system is thought to bridge innate immunity and subsequent adaptive immune responses.

In addition to IL-27, other T helper cells populations can also negatively regulate the development of T_H_17 cells. As mentioned earlier, IFN-*γ* produced by T_H_1 cells upregulates the T-bet transcriptional factor which dampens the activation of ROR*γ*t resulting in the downregulation of T_H_17 cells. Similarly, the upregulation of GATA-3 transcription factor of T_H_2 cells by IL-4/5/13 could also restrict the expansion of T_H_17 cells by inhibiting the function of ROR*γ*t. One major aspect of   T_H_17 cells negative regulation is the influence of Treg cells. Treg cell differentiation is driven mainly by TGF-*β* which activates Foxp3. Sharing the ubiquitous TGF-*β* factor, the presence or absence of IL-6 controls the developmental shift toward T_H_17 or Treg cells. The shift to CD4+CD25+Foxp3+ Treg and CD4+CD25+Foxp3+CD39+ subset plays a significant role in restricting the detrimental effect of T_H_17 cells in multiple sclerosis patients [74]. Interestingly, retinoic acid increases the expression of Foxp3 via activation and phosphorylation of Smad3 and concomitantly inhibits the expression of IL-6R*α*, IRF-4, and IL-23R, thereby limiting T_H_17 development [75]. An exciting and confounding feature of T_H_17 development is the plasticity among different T helper cells populations and the microenvironment or microflora that imposes on its lineage-specific differentiation. A study by Koenen et al. has demonstrated that human CD25highFoxp3+ Treg cells when stimulated with allogeneic monocytes in the presence of IL-2 and IL-15 can differentiate into IL-17 producing T cells. The study further showed that the lateral lineage conversion to T_H_17 cells from Treg cells relied on the histone deacetylase activity indicating the contribution of epigenetic modification [76]. In addition, T_H_17 cells have the propensity to convert to T_H_17/T_H_1 phenotype under the appropriate milieu of low TGF-*β* and high IL-12 levels which are often observed in the joints of children with inflammatory arthritis [77]. Other microorganisms such as live *C. albicans *can modulate tryptophan metabolism to inhibit IL-17 production [78], and *H. pylori *mediates the polarization of T_H_17/Treg balance toward regulatory response which inhibits T_H_17 response [79].

## 4. IL-17 in Murine Lupus

As previously mentioned, the role of T_H_17 in the development of autoimmunity was initially scrutinized in murine models of induced EAE [2]. This disease model was originally believed to be dependent on IL-12, and thus, T_H_1 mediated. However, the revelation that IL-12 shared a subunit, p40, with a newly discovered cytokine, IL-23, and that this novel cytokine, not IL-12, was required for induction of disease sets the stage for investigation of T_H_17 these models [4, 5]. More recently, several lines of research have reported increased IL-17 production and T_H_17 functions in murine models of lupus as summarized in Table 1. 

BXD2 is one of 20 BXD recombinant inbred strains derived from a cross between C57BL/6J (B6) and DBA/2J. These mice develop a spontaneous and age-dependent lupus-like syndrome denoted by production of the canonical anti-DNA, antihistone, and rheumatoid factor autoantibodies, as well as splenomegaly, glomerulonephritis (GN), and erosive arthritis [80, 81]. BXD2 CD4+ T cells have enhanced T_H_17 development and consequent increased serum levels of IL-17 [82]. Moreover, IL-17-secreting CD4+ cells were shown to localize to germinal centers (GCs) in BXD2 spleens. This augmented IL-17 response was associated with increased GC development and stability in BXD2 spleens as compared to B6 controls. Additionally, BXD2 have increased amounts of IL-17R+ B cells [82]. These B cells have both an increased basal and an IL-17R-induced activation of the canonical NF*κβ* pathway, resulting in an increased expression of regulator of G signaling (RGS) proteins [83]. Consequently, RGSs enhance the GTPase activity of chemokine receptor G*α* subunits resulting in decreased chemotaxis [84, 85]. Indeed, BXD2 B cells were shown to have a diminished chemotactic response to CXCL12 and CXCL13, especially in the presence of IL-17 [82, 83]. This increased potential for B cell accumulation at the sites of CXCL12 and CXCL13 production, such as follicular dendritic cell rich areas [86, 87], is the likely cause of the enhanced GC formation in the BDX2 strain. Moreover, the concurrent production of IL-17 by T_H_17 cells in GCs further promotes B cell accumulation and GC stability. IL-17 also results in increased activation-induced cytidine deaminase (*Aicda*) expression and somatic hypermutation in BXD2 IL-17R+ B cells, which have an intrinsically enhanced ability to produce autoantibodies as compared to IL-17R-deficient BDX2 B cells [82]. Thus IL-17 has a central role in pathogenesis of the lupus-like syndrome observed in this model. 

The MRL/*lpr* strain is a classical model of spontaneous lupus. It exhibits a lymphoproliferative disorder which manifests with autoantibody production, GN, and accumulation of CD4^−^CD8^−^ double-negative T (DNT) cells in the periphery [88]. A mutation in *Fas* is responsible for the *lpr* phenotype and is the major functional contributor of pathogenesis in this strain [89, 90]. It was recently shown that *Fas*-deficient DNT cells are capable of producing significant amounts of IL-17 [91]. Further, the T_H_17-stablizing cytokine, IL-23, potently induced IL-17 production in these DNT cells which were then capable of renal infiltration and GN induction. Finally, deletion of IL-23R prevented splenomegaly, lymphadenopathy, autoantibody production, and GN in the context of *Fas* deficiency and was associated with a major reduction of the DNT cell compartment along with its concomitant IL-17 production [92]. Thus, a pathogenic T_H_17-like function of DNT cells has been exposed, highlighting this subset as a target for disease intervention. 

The SNF1 mouse model, derived from the F1 outcross of the New Zealand Black and SWR recombinant inbred strains, develops a spontaneous lupus-like syndrome that can be accelerated by immunization of nucleosomal peptides [93]. Upon disease induction, autoantibodies are produced, and GN with T_H_17 infiltration is initiated [94]. Interestingly, low-dose therapy of a tolerogenic histone-derived peptide caused increased TGF-*β* and decreased IL-6 expression in dendritic cells and resulted in enhancement of Treg function with a reduction in T_H_17 renal infiltrates [94]. Treatment with either oral or nasal anti-CD3 also ameliorates autoantibody production and nephritis in this model by inducing a regulatory T cell subset and reducing IL-17 production by T follicular helper cells [95, 96]. These results indicate that therapies that regulate Treg/T_H_17 homeostasis in favor of Treg might be effective at moderating SLE pathogenesis.

Finally, disruption of TNF*α* receptor signaling in spontaneous lupus-prone NZM2328 mice results in exacerbated disease that has associated with a greatly enhanced T effector/memory compartment. These cells were found to have a Th17 gene signature and produced more IL-17 than TNF-*α* receptor sufficient T effector/memory cells [97]. This work highlights the regulatory function that TNF-*α* can have and sets a caution for TNF-*α* blockade therapy.

## 5. IL-17 in SLE Patients

As with murine lupus models, evidence for a T_H_17 role in human SLE is also mounting. Several recent reports show that plasma IL-17 and IL-17 producing T cells are increased in SLE patients [29–34]. Moreover, disease activity and severity are associated with increased IL-17 production [31–34]. SLE patients have increased phosphorylation of STAT3 [98], which is required for T_H_17 differentiation, as STAT3 deficiency in hyper-IgE syndrome patients results in the ablation of T_H_17 cells [99, 100]. The T_H_17-polarizing cytokines, IL-6, IL-21, and IL-23, all signal in a STAT3-dependent manner to induce transcription of the ROR*γ*t [101]. Indeed, SLE patients also have increased plasma levels of IL-6, and higher *Rorc* expression, which encodes ROR*γ*t [34, 102]. Taken together, T_H_17 expansion is an important feature of SLE that needs to be further investigated. 

It is well established that there is a strong gender bias in the incidence of SLE in which roughly 90% of the cases occur in females. Since IL-17 production correlates with disease severity, the question is raised as to whether the female bias of SLE is due to differences in T_H_17 biology. While this has not been studied extensively, IL-17 *in vitro *production was shown to decrease with age in males, but not in females [103]. Although these results do demonstrate a gender difference, the relevance to SLE induction is not clear since the young cohorts, who were between 21 and 40 years old, the highly susceptible age of onset for SLE, did not produce different amounts of IL-17 in males versus females. Nevertheless, the ability to maintain higher levels of IL-17 production with age may contribute to the maintenance of the disease state in females. More recently, it was reported that *in vivo *treatment of mice with estrogen enhances T_H_17 polarization *in vitro*, supporting the hypothesis that T_H_17 cells contribute to the female bias of SLE [104]. There is, however, no direct evidence for this hypothesis, and further study is needed to clarify the role that gender may play in T_H_17 function and disease induction.

Similar to *Fas*-deficient mouse models of lupus, a significant amount of IL-17 is also produced by an expanded subset of DNT cells in SLE patients [30]. These DNT cells are derived from CD8^+^ cells that have downregulated CD8 in response to receptor stimulation [105]. While they are normally present in very small amounts, their expansion in SLE patients may be due to increased T cell activation. Because of their downregulated coreceptor, they have decreased survival and proliferation and display unique gene expression patterns and proinflammatory cytokine profiles [105]. Notably, as in lupus-prone mice, DNT cells can be found in kidney biopsies of SLE patients [30]. Therefore, DNT cells appear to represent a distinct effector population of T cells whose dysregulation may be central to SLE pathogenesis.

The fundamental role of type I IFN dysregulation is well established in SLE pathogenesis [106]. Unregulated IFN-*α* production has been shown to increase proinflammatory cytokine production, including IL-6 and IL-23 which lead to Th17-mediated inflammation in mice [107]. Also plasmacytoid dendritic cells (pDCs), which are known to potently secrete IFN-*α*, also produce IL-1*β*, IL-6, and IL-23 in response to Toll-like receptor (TLR)-7 stimulation in human studies [108, 109]. These pDCs are capable of inducing T_H_17 differentiation when cocultured with CD4^+^ cells. Endogenous nucleic acids are autoantibody targets in SLE and are capable of TLR activation following their uptake as immune complexes [110, 111]. Therefore, pDCs can be chronically activated, potentiating Th17 development and disease pathogenesis. 

**Figure 1:**
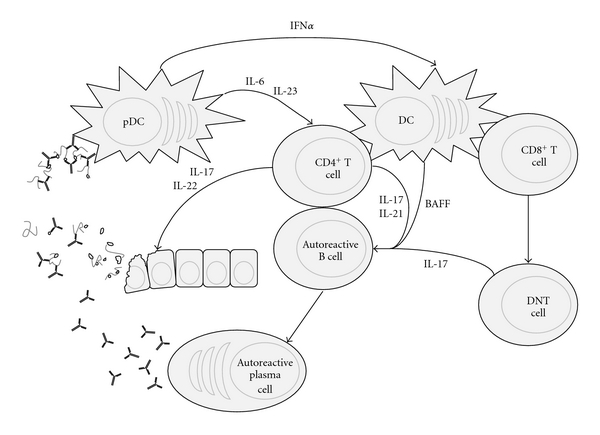
IL-17 in SLE pathogenesis. IL-17, IL-21, and BAFF promote survival, class-switching, and production of antinuclear autoantibodies by autoreactive B cells. Consequently, nucleic acid-containing immune complexes stimulate plasmacytoid dendritic cells (pDCs) to produce type I interferon, IL-6, and IL-23, which enhance DC activation, and T_H_17 induction, thus completing a feedback loop for autoimmune activation. Concurrently, hyperactivation in the context of autoimmunity may actuate the accumulation of double-negative T (DNT) cells which produce more IL-17 and exacerbates the disease state. Ultimately, T_H_17 and DNT cells infiltrate systemic tissues and incite end organ disease.

IL-17 also promotes B cell survival both alone and synergistically with B cell-activating factor (BAFF) [31]. Hence, a feedback loop is established where IL-17 promotes autoreactive B cells to persist longer and make autoantibodies which activate pDCs induce more T_H_17 cells. In parallel, the expansion of DNT cells results in more IL-17 production, exacerbating this progression (Figure 1). As IL-17 is central mediator to this process, therapeutic intervention that targets T_H_17 development and IL-17 production will be valuable treatments for SLE.

## 6. Conclusions

The discovery of IL-17 and T_H_17 cells has expanded and transformed the conventional thought in immunology. The change adds complexity to an already complicated matter. The intricate and dynamic interaction between the different characters promotes the adaptability and resiliency of the immune system. Therefore, it is difficult to comprehend that any one particular entity is solely responsible for such a vexing system. The coincidental discovery of T_H_17 cells did not shift any paradigms, but merely add another unknown factor to an unsolved equation. Currently, there are numbers of issues that need to be resolved, for example, the differential function of IL-17 molecules within the family in the context of infectious disease and autoimmunity, the negative regulation of T_H_17 cells and its application in therapeutic approach, and its relevance in the pathogenesis of SLE besides observational or correlative studies. Tremendous strides are being made to address these issues.

## Figures and Tables

**Table 1:** IL-17 in murine models of lupus.

Model	Description	References
BXD2	IL-17 promotes spontaneous GC development as well as autoantibody production by IL-17R^+^ B cells	[82, 83]
MRL.*lpr *	Expansion of IL-17-producing DNT cells with kidney infiltration and GN induction	[91, 92]
SNF1	Enhanced IL-17 production by CD4^+^ T cells with kidney infiltration	[94]
NZM2328	Disruption of TNF*α* promotes Th17 development	[97]

## References

[1] Kastelein RA, Hunter CA, Cua DJ (2007). Discovery and biology of IL-23 and IL-27: related but functionally distinct regulators of inflammation. *Annual Review of Immunology*.

[2] Korn T, Bettelli E, Oukka M, Kuchroo VK (2009). IL-17 and Th17 cells. *Annual Review of Immunology*.

[3] Stockinger B, Veldhoen M, Martin B (2007). Th17 T cells: linking innate and adaptive immunity. *Seminars in Immunology*.

[4] Cua DJ, Sherlock J, Chen Y (2003). Interleukin-23 rather than interleukin-12 is the critical cytokine for autoimmune inflammation of the brain. *Nature*.

[5] Murphy CA, Langrish CL, Chen Y (2003). Divergent pro- and antiinflammatory roles for IL-23 and IL-12 in joint autoimmune inflammation. *Journal of Experimental Medicine*.

[6] Harrington LE, Hatton RD, Mangan PR (2005). Interleukin 17-producing CD4^+^ effector T cells develop via a lineage distinct from the T helper type 1 and 2 lineages. *Nature Immunology*.

[7] Park H, Li Z, Yang XO (2005). A distinct lineage of CD4 T cells regulates tissue inflammation by producing interleukin 17. *Nature Immunology*.

[8] Veldhoen M, Hocking RJ, Atkins CJ, Locksley RM, Stockinger B (2006). TGF*β* in the context of an inflammatory cytokine milieu supports de novo differentiation of IL-17-producing T cells. *Immunity*.

[9] Bettelli E, Carrier Y, Gao W (2006). Reciprocal developmental pathways for the generation of pathogenic effector T_H_17 and regulatory T cells. *Nature*.

[10] Mangan PR, Harrington LE, O’Quinn DB (2006). Transforming growth factor-*β* induces development of the T_H_17 lineage. *Nature*.

[11] Hunter CA (2005). New IL-12-family members: IL-23 and IL-27, cytokines with divergent functions. *Nature Reviews Immunology*.

[12] Ivanov II, McKenzie BS, Zhou L (2006). The orphan nuclear receptor ROR*γ*t directs the differentiation program of proinflammatory IL-17^+^ T helper cells. *Cell*.

[13] Manel N, Unutmaz D, Littman DR (2008). The differentiation of human T_H_17 cells requires transforming growth factor-*β* and induction of the nuclear receptor ROR*γ*t. *Nature Immunology*.

[14] Okamoto K, Iwai Y, Oh-Hora M (2010). I*κ*B*η* regulates T_H_17 development by cooperating with ROR nuclear receptors. *Nature*.

[15] Schraml BU, Hildner K, Ise W (2009). The AP-1 transcription factor Batf controls T_H_17 differentiation. *Nature*.

[16] Chen Q, Yang W, Gupta S (2008). IRF-4-binding protein inhibits interleukin-17 and interleukin-21 production by controlling the activity of IRF-4 transcription factor. *Immunity*.

[17] Zhang F, Meng G, Strober W (2008). Interactions among the transcription factors Runx1, ROR*γ*t and Foxp3 regulate the differentiation of interleukin 17-producing T cells. *Nature Immunology*.

[18] Gaffen SL (2009). Structure and signalling in the IL-17 receptor family. *Nature Reviews Immunology*.

[19] Ely LK, Fischer S, Garcia KC (2009). Structural basis of receptor sharing by interleukin 17 cytokines. *Nature Immunology*.

[20] Gerhardt S, Abbott WM, Hargreaves D (2009). Structure of IL-17A in complex with a potent, fully human neutralizing antibody. *Journal of Molecular Biology*.

[21] Hymowitz SG, Filvaroff EH, Yin J (2001). IL-17s adopt a cystine knot fold: structure and activity of a novel cytokine, IL-17F, and implications for receptor binding. *The EMBO Journal*.

[22] Weaver CT, Hatton RD, Mangan PR, Harrington LE (2007). IL-17 family cytokines and the expanding diversity of effector T cell lineages. *Annual Review of Immunology*.

[23] Ishigame H, Kakuta S, Nagai T (2009). Differential roles of interleukin-17A and -17F in host defense against mucoepithelial bacterial infection and allergic responses. *Immunity*.

[24] Bettelli E, Korn T, Oukka M, Kuchroo VK (2008). Induction and effector functions of T_H_17 cells. *Nature*.

[25] Duerr RH, Taylor KD, Brant SR (2006). A genome-wide association study identifies IL23R as an inflammatory bowel disease gene. *Science*.

[26] Hue S, Ahern P, Buonocore S (2006). Interleukin-23 drives innate and T cell-mediated intestinal inflammation. *Journal of Experimental Medicine*.

[27] Nguyen CQ, Hu MH, Li Y, Stewart C, Peck AB (2008). Salivary gland tissue expression of interleukin-23 and interleukin-17 in Sjögren’s syndrome: findings in humans and mice. *Arthritis and Rheumatism*.

[28] Nguyen CQ, Yin H, Lee BH, Chiorini JA, Peck AB (2011). IL17: potential therapeutic target in Sjögren's syndrome using adenovirus-mediated gene transfer. *Laboratory Investigation*.

[29] Mok MY, Wu HJ, Lo Y, Lau CS (2010). The relation of interleukin 17 (IL-17) and IL-23 to Th1/Th2 cytokines and disease activity in systemic lupus erythematosus. *Journal of Rheumatology*.

[30] Crispín JC, Oukka M, Bayliss G (2008). Expanded double negative T cells in patients with systemic lupus erythematosus produce IL-17 and infiltrate the kidneys. *Journal of Immunology*.

[31] Doreau A, Belot A, Bastid J (2009). Interleukin 17 acts in synergy with B cell-activating factor to influence B cell biology and the pathophysiology of systemic lupus erythematosus. *Nature Immunology*.

[32] Shah K, Lee W-W, Lee S-H (2010). Dysregulated balance of Th17 and Th1 cells in systemic lupus erythematosus. *Arthritis Research and Therapy*.

[33] Wong CK, Lit LCW, Tam LS, Li EKM, Wong PTY, Lam CWK (2008). Hyperproduction of IL-23 and IL-17 in patients with systemic lupus erythematosus: implications for Th17-mediated inflammation in auto-immunity. *Clinical Immunology*.

[34] Chen XQ, Yu YC, Deng HH (2010). Plasma IL-17A is increased in new-onset SLE patients and associated with disease activity. *Journal of Clinical Immunology*.

[35] Toy D, Kugler D, Wolfson M (2006). Cutting edge: interleukin 17 signals through a heteromeric receptor complex. *Journal of Immunology*.

[36] Dubin PJ, Kolls JK (2009). Interleukin-17A and interleukin-17F: a tale of two cytokines. *Immunity*.

[37] Yamaguchi Y, Fujio K, Shoda H (2007). IL-17B and IL-17C are associated with TNF-*α* production and contribute to the exacerbation of inflammatory arthritis. *Journal of Immunology*.

[38] Ryan AW, Thornton JM, Brophy K (2005). Chromosome 5q candidate genes in coeliac disease: genetic variation at IL4, IL5, IL9, IL13, IL17B and NR3C1. *Tissue Antigens*.

[39] Fort MM, Cheung J, Yen D (2001). IL-25 Induces IL-4, IL-5, and IL-13 and Th2-associated pathologies in vivo. *Immunity*.

[40] Cheung PFY, Wong CK, Ip WK, Lam CWK (2006). IL-25 regulates the expression of adhesion molecules on eosinophils: mechanism of eosinophilia in allergic inflammation. *Allergy*.

[41] Caruso R, Stolfi C, Sarra M (2009). Inhibition of monocyte-derived inflammatory cytokines by IL-25 occurs via p38 Map kinase-dependent induction of Socs-3. *Blood*.

[42] Ballantyne SJ, Barlow JL, Jolin HE (2007). Blocking IL-25 prevents airway hyperresponsiveness in allergic asthma. *Journal of Allergy and Clinical Immunology*.

[43] Rickel EA, Siegel LA, Bo-Rin PY (2008). Identification of functional roles for both IL-17RB and IL-17RA in mediating IL-25-induced activities. *Journal of Immunology*.

[44] Kleinschek MA, Owyang AM, Joyce-Shaikh B (2007). IL-25 regulates Th17 function in autoimmune inflammation. *Journal of Experimental Medicine*.

[45] Büning C, Genschel J, Weltrich R, Lochs H, Schmidt H (2003). The interleukin-25 gene located in the inflammatory bowel disease (IBD) 4 region: no association with inflammatory bowel disease. *European Journal of Immunogenetics*.

[46] Korn T, Bettelli E, Gao W (2007). IL-21 initiates an alternative pathway to induce proinflammatory T_H_17 cells. *Nature*.

[47] Huber M, Brüstle A, Reinhard K (2008). IRF4 is essential for IL-21-mediated induction, amplification, and stabilization of the Th17 phenotype. *Proceedings of the National Academy of Sciences of the United States of America*.

[48] Kuchen S, Robbins R, Sims GP (2007). Essential role of IL-21 in B cell activation, expansion, and plasma cell generation during CD4^4^ T cell-B cell collaboration. *Journal of Immunology*.

[49] Pène J, Gauchat JF, Lécart S (2004). Cutting edge: IL-21 is a switch factor for the production of IgG_1_ and IgG_3_ by human B cells. *Journal of Immunology*.

[50] Sarra M, Monteleone G (2010). Interleukin-21: a new mediator of inflammation in systemic lupus erythematosus. *Journal of Biomedicine and Biotechnology*.

[51] Nguyen CQ, Gao JH, Kim H, Saban DR, Cornelius JG, Peck AB (2007). IL-4-STAT6 signal transduction-dependent induction of the clinical phase of Sjögren’s syndrome-like disease of the nonobese diabetic mouse. *Journal of Immunology*.

[52] Brayer JB, Cha S, Nagashima H (2001). IL-4-dependent effector phase in autoimmune exocrinopathy as defined by the NOD.IL-4-gene knockout mouse model of Sjögren’s syndrome. *Scandinavian Journal of Immunology*.

[53] Gao J, Killedar S, Cornelius JG, Nguyen C, Cha S, Peck AB (2006). Sjögren’s syndrome in the NOD mouse model is an interleukin-4 time-dependent, antibody isotype-specific autoimmune disease. *Journal of Autoimmunity*.

[54] Gao J, Cha S, Jonsson R, Opalko J, Peck AB (2004). Detection of anti-type 3 muscarinic acetylcholine receptor autoantibodies in the sera of Sjögren’s syndrome patients by use of a transfected cell line assay. *Arthritis and Rheumatism*.

[55] Bauquet AT, Jin H, Paterson AM (2009). The costimulatory molecule ICOS regulates the expression of c-Maf and IL-21 in the development of follicular T helper cells and T_H_-17 cells. *Nature Immunology*.

[56] Kotenko SV, Izotova LS, Mirochnitchenko OV (2001). Identification of the functional interleukin-22 (IL-22) receptor complex. The IL-10R2 chain (IL-10R*β*) is a common chain of both the IL-10 and IL-22 (IL-10-related T cell-derived inducible factor, IL-TIF) receptor complexes. *Journal of Biological Chemistry*.

[57] Xie MH, Aggarwal S, Ho WH (2000). Interleukin (IL)-22, a novel human cytokine that signals through the interferon receptor-related proteins CRF2-4 and IL-22R. *Journal of Biological Chemistry*.

[58] Lejeune D, Dumoutier L, Constantinescu S, Kruijer W, Schuringa JJ, Renauld JC (2002). Interleukin-22 (IL-22) activates the JAK/STAT, ERK, JNK, and p38 MAP kinase pathways in a rat hepatoma cell line: pathways that are shared with and distinct from IL-10. *Journal of Biological Chemistry*.

[59] Ouyang W, Kolls JK, Zheng Y (2008). The biological functions of T helper 17 cell effector cytokines in inflammation. *Immunity*.

[60] Vivier E, Spits H, Cupedo T (2009). Interleukin-22-producing innate immune cells: new players in mucosal immunity and tissue repair?. *Nature Reviews Immunology*.

[61] Ikeuchi H, Kuroiwa T, Hiramatsu N (2005). Expression of interleukin-22 in rheumatoid arthritis: potential role as a proinflammatory cytokine. *Arthritis and Rheumatism*.

[62] Brand S, Beigel F, Olszak T (2006). IL-22 is increased in active Crohn’s disease and promotes proinflammatory gene expression and intestinal epithelial cell migration. *American Journal of Physiology*.

[63] Whittington HA, Armstrong L, Uppington KM, Millar AB (2004). Interleukin-22: a potential immunomodulatory molecule in the lung. *American Journal of Respiratory Cell and Molecular Biology*.

[64] Aujla SJ, Chan YR, Zheng M (2008). IL-22 mediates mucosal host defense against Gram-negative bacterial pneumonia. *Nature Medicine*.

[65] Yoshida H, Yoshiyuki M (2008). Regulation of immune responses by interleukin-27. *Immunological Reviews*.

[66] Pflanz S, Timans JC, Cheung J (2002). IL-27, a heterodimeric cytokine composed of EBI3 and p28 protein, induces proliferation of naive CD4^+^ T cells. *Immunity*.

[67] Villarino AV, Huang E, Hunter CA (2004). Understanding the pro- and anti-inflammatory properties of IL-27. *Journal of Immunology*.

[68] D’Acquisto F, Maione F, Pederzoli-Ribeil M (2010). From IL-15 to IL-33: the never-ending list of new players in inflammation. Is it time to forget the humble aspirin and move ahead?. *Biochemical Pharmacology*.

[69] Yoshida H, Nakaya M, Miyazaki Y (2009). Interleukin 27: a double-edged sword for offense and defense. *Journal of Leukocyte Biology*.

[70] Seita J, Asakawa M, Ooehara J (2008). Interleukin-27 directly induces differentiation in hematopoietic stem cells. *Blood*.

[71] Nagai H, Oniki S, Fujiwara S (2010). Antitumor hiroshi nagai activities of interleukin-27 on melanoma. *Endocrine, Metabolic & Immune Disorders Drug Targets*.

[72] Yoshimura T, Takeda A, Hamano S (2006). Two-sided roles of IL-27: induction of Th1 differentiation on naive CD4^+^ T cells versus suppression of proinflammatory cytokine production including IL-23-induced IL-17 on activated CD4^+^ T cells partially through STAT3-dependent mechanism. *Journal of Immunology*.

[73] Amadi-Obi A, Yu CR, Liu X (2007). T_H_17
cells contribute to uveitis and scleritis and are expanded by IL-2 and inhibited by IL-27/STAT1. *Nature Medicine*.

[74] Fletcher JM, Lonergan R, Costelloe L (2009). CD39^+^Foxp3^+^ regulatory T cells suppress pathogenic Th17 cells and are impaired in multiple sclerosis. *Journal of Immunology*.

[75] Xiao S, Jin H, Korn T (2008). Retinoic acid increases Foxp3^+^ regulatory T cells and inhibits development of Th17 cells by enhancing TGF-*β*-driven Smad3 signaling and inhibiting IL-6 and IL-23 receptor expression. *Journal of Immunology*.

[76] Koenen HJPM, Smeets RL, Vink PM, van Rijssen E, Boots AMH, Joosten I (2008). Human CD25^high^Foxp3^pos^ regulatory T cells differentiate into IL-17 producing cells. *Blood*.

[77] Nistala K, Adams S, Cambrook H (2010). Th17 plasticity in human autoimmune arthritis is driven by the inflammatory environment. *Proceedings of the National Academy of Sciences of the United States of America*.

[78] Cheng S-C, van de Veerdonk F, Smeekens S (2010). Candida albicans Dampens host defense by downregulating IL-17 production. *Journal of Immunology*.

[79] Kao JY, Zhang M, Miller MJ (2010). Helicobacter pylori immune escape is mediated by dendritic cell-induced Treg skewing and Th17 suppression in mice. *Gastroenterology*.

[80] Mountz JD, Yang P, Wu Q (2005). Genetic segregation of spontaneous erosive arthritis and generalized autoimmune disease in the BXD2 recombinant inbred strain of mice. *Scandinavian Journal of Immunology*.

[81] Hsu HC, Zhou T, Kim H (2006). Production of a novel class of polyreactive pathogenic autoantibodies in BXD2 mice causes glomerulonephritis and arthritis. *Arthritis and Rheumatism*.

[82] Hsu HC, Yang PA, Wang J (2008). Interleukin 17-producing T helper cells and interleukin 17 orchestrate autoreactive germinal center development in autoimmune BXD2 mice. *Nature Immunology*.

[83] Xie S, Li J, Wang JH (2010). IL-17 activates the canonical NF-*κ*B signaling pathway in autoimmune B cells of BXD2 mice to upregulate the expression of regulators of G-protein signaling 16. *Journal of Immunology*.

[84] Koelle MR (1997). A new family of G-protein regulators—the RGS proteins. *Current Opinion in Cell Biology*.

[85] Shi GX, Harrison K, Wilson GL, Moratz C, Kehrl JH (2002). RGS13 regulates germinal center B lymphocytes responsiveness to CXC chemokine ligand (CXCL)12 and CXCL13. *Journal of Immunology*.

[86] Allen CDC, Ansel KM, Low C (2004). Germinal center dark and light zone organization is mediated by CXCR4 and CXCR5. *Nature Immunology*.

[87] Allen CDC, Okada T, Cyster JG (2007). Germinal-center organization and cellular dynamics. *Immunity*.

[88] Cohen PL, Eisenberg RA (1991). Lpr and gld: single gene models of systemic autoimmunity and lymphoproliferative disease. *Annual Review of Immunology*.

[89] Adachi M, Watanabe-Fukunaga R, Nagata S (1993). Aberrant transcription caused by the insertion of an early transposable element in an intron of the Fas antigen gene of lpr mice. *Proceedings of the National Academy of Sciences of the United States of America*.

[90] Kono DH, Theofilopoulos AN (2006). Genetics of SLE in mice. *Springer Seminars in Immunopathology*.

[91] Zhang Z, Kyttaris VC, Tsokos GC (2009). The role of IL-23/IL-17 axis in lupus nephritis. *Journal of Immunology*.

[92] Kyttaris VC, Zhang Z, Kuchroo VK, Oukka M, Tsokos GC (2010). Cutting edge: IL-23 receptor deficiency prevents the development of lupus nephritis in C57BL/6-lpr/lpr mice. *Journal of Immunology*.

[93] Kaliyaperumal A, Mohan C, Wu W, Datta SK (1996). Nucleosomal peptide epitopes for nephritis-inducing T helper cells of murine lupus. *Journal of Experimental Medicine*.

[94] Kang HK, Liu M, Datta SK (2007). Low-dose peptide tolerance therapy of lupus generates plasmacytoid dendritic cells that cause expansion of autoantigen-specific regulatory T cells and contraction of inflammatory Th17 cells. *Journal of Immunology*.

[95] Wu HY, Quintana FJ, Weiner HL (2008). Nasal anti-CD3 antibody ameliorates lupus by inducing an IL-10-secreting CD4^+^CD25^−^LAP^+^ regulatory T Cell and is associated with down-regulation of IL-17^+^CD4^+^ICOS^+^CXCR5^+^ follicular helper T cells. *Journal of Immunology*.

[96] Wu HY, Center EM, Tsokos GC, Weiner HL (2009). Suppression of murine SLE by oral anti-CD3: inducible CD4^+^CD25^−^LAP^+^ regulatory T cells control the expansion of IL-17^+^ follicular helper T cells. *Lupus*.

[97] Jacob N, Yang H, Pricop L (2009). Accelerated pathological and clinical nephritis in systemic lupus erythematosus-prone New Zealand mixed 2328 mice doubly deficient in TNF receptor 1 and TNF receptor 2 via a Th17-associated pathway. *Journal of Immunology*.

[98] Harada T, Kyttaris V, Li Y, Juang YT, Wang Y, Tsokos GC (2007). Increased expression of STAT3 in SLE T cells contributes to enhanced chemokine-mediated cell migration. *Autoimmunity*.

[99] Ma CS, Chew GYJ, Simpson N (2008). Deficiency of Th17 cells in hyper IgE syndrome due to mutations in STAT3. *Journal of Experimental Medicine*.

[100] de Beaucoudtey L, Puel A, Filipe-Santos O (2008). Mutations in STAT3 and IL12RB1 impair the development of human IL-17-producing T cells. *Journal of Experimental Medicine*.

[101] Chen Z, Laurence A, O’Shea JJ (2007). Signal transduction pathways and transcriptional regulation in the control of Th17 differentiation. *Seminars in Immunology*.

[102] Chun HY, Chung JW, Kim HA (2007). Cytokine IL-6 and IL-10 as biomarkers in systemic lupus erythematosus. *Journal of Clinical Immunology*.

[103] Goetzl EJ, Huang M-C, Kon J (2010). Gender specificity of altered human immune cytokine profiles in aging. *FASEB Journal*.

[104] Khan D, Dai R, Karpuzoglu E, Ahmed SA (2010). Estrogen increases, whereas IL-27 and IFN-*γ* decrease, splenocyte IL-17 production in WT mice. *European Journal of Immunology*.

[105] Crispín JC, Tsokos GC (2009). Human TCR-*αβ*
^+^CD4^−^CD8^−^ T cells can derive from CD8^+^ T cells and display an inflammatory effector phenotype. *Journal of Immunology*.

[106] Pascual V, Farkas L, Banchereau J (2006). Systemic lupus erythematosus: all roads lead to type I interferons. *Current Opinion in Immunology*.

[107] Espinosa A, Dardalhon V, Brauner S (2009). Loss of the lupus autoantigen Ro52/Trim21 induces tissue inflammation and systemic autoimmunity by disregulating the IL-23-Th17 pathway. *Journal of Experimental Medicine*.

[108] Lombardi V, van Overtvelt L, Horiot S, Moingeon P (2009). Human dendritic cells stimulated via TLR7 and/or TLR8 induce the sequential production of Il-10, IFN-*γ*, and IL-17A by naive CD4^+^ T cells. *Journal of Immunology*.

[109] Yu CF, Peng WM, Oldenburg J (2010). Human plasmacytoid dendritic cells support Th17 cell effector function in response to TLR7 ligation. *Journal of Immunology*.

[110] Barrat FJ, Meeker T, Gregorio J (2005). Nucleic acids of mammalian origin can act as endogenous ligands for Toll-like receptors and may promote systemic lupus erythematosus. *Journal of Experimental Medicine*.

[111] Vollmer J, Tluk S, Schmitz C (2005). Immune stimulation mediated by autoantigen binding sites within small nuclear RNAs involves Toll-like receptors 7 and 8. *Journal of Experimental Medicine*.

